# Torsional Abnormality: The Forgotten Issue in the Diagnosis and Treatment of the Anterior Knee Pain Patient

**DOI:** 10.3390/jcm11123530

**Published:** 2022-06-20

**Authors:** Vicente Sanchis-Alfonso, Robert A. Teitge

**Affiliations:** 1 Department of Orthopedic Surgery, Hospital Arnau de Vilanova, 46015 Valencia, Spain; 2 Department of Orthopaedic Surgery, Wayne State University, Detroit, MI 48202, USA; rteitge@hotmail.com

Currently, no one doubts that the vast majority of anterior knee pain (AKP) cases do not need surgery [1,2]. What is more, it is widely accepted that in more cases than is acceptable, the AKP worsens after surgical treatment [2]. However, only 60% of AKP patients have a satisfactory outcome after conservative treatment at 12 months after the initial diagnosis [3]. Something does not add up here. This very low success rate for conservative treatment may be due to the fact that some of these patients are not really candidates for conservative treatment. Perhaps we are overlooking causes of AKP in which surgery might be the only reasonable treatment of choice. Torsional abnormality in the limb is one such cause. No one doubts that AKP etiopathogenesis is multifactorial [2]. Therefore, there are several subsets of AKP patients and therefore several types of treatments [2].

In this Special Issue, we focus on skeletal malalignment, and especially on torsional abnormalities of the lower limb, because abnormal limb torsion is probably the most overlooked factor in patellofemoral joint pathology, both in the genesis of pain, but even more so in the treatment. Unfortunately, the vast majority of orthopedic surgeons consider that patellar malalignment, that is, patellar tilt and/or patellar shift, is a problem of the patella on the trochlea. In reality, patellar malalignment is often the direct result of an abnormal limb alignment. We must always consider that limb malalignment, not patellar malalignment, might be the real etiology [4]. Limb malalignment may exist in any of the three planes, but the transverse plane alignment, torsion, is the most overlooked [5]. Thus, it is of vital importance to assess the rotational profiles of the femur and tibia in an AKP patient. As far back as 1995, Flandry and Hughston showed that the most frequent cause of failure of an extensor mechanism realignment surgery is the existence of an underlying torsional abnormality that is not diagnosed and therefore is not treated [6]. 

At the end of 1970s, skeletal malalignment of the lower limb was suggested as one of the causes of AKP in some young patients [7]. Skeletal malalignment is defined as the malalignment of the limb measured on the transverse, coronal, and sagittal planes. In particular, rotational abnormalities are important [5,8]. However, the concept of skeletal malalignment has had extremely little influence on orthopedic surgeons even until today. In fact, very few publications refer to skeletal malalignment as a cause of AKP. From 1990 to June 2021, we could only find 22 published papers analyzing, from a clinical point of view, the association between patellofemoral disorders in young patients and torsional abnormalities of the femur and/or tibia [9]. These data lead us to ask the following question. Why do we ignore torsional abnormalities in the diagnosis and especially in the treatment of AKP patients? 

The main reason is the diagnostic uncertainty. Limb alignment on the transverse plane is hard to see and difficult to measure. Currently, there is no consensus on how to measure torsion [10,11], and we must note that accurate measurement of torsion is essential to diagnosis, correct surgical decision-making, and preoperative planning of a rotational osteotomy (i.e., the amount of correction needed). 

The first problem that is faced when we see a patient with a torsional abnormality is to recognize it and then to measure it [10,11]. Recognition and measurement of torsion by physical examination is somewhat subjective and unreliable. There are 28 methods to measure femoral anteversion (FAV) [10]. Mean values reported in the literature range from 5.8° to 24.1°. Murphy et al. [12] have shown that the traditional methods used to measure FAV may underestimate the actual FAV by a mean 13° and as much as 18°. In the same way, Kaiser et al. [13] have shown a significant difference in measurement techniques of up to 100% (11°–22°). The CT method that we use to evaluate FAV is the one described by Murphy in 1987 [12]. Murphy’s method comes closest to defining reality, as it started with the physical measurement of anatomic specimens. His method of anteversion measurement correlates well with physical examination. Interestingly, Schmaranzer et al. [14] have observed that the differences between the classic and Murphy’s method [12] become more evident in patients with a clinical diagnosis of FAV. It has been shown that the difference in FAV between the classic method [15] and Murphy’s method [12] increased from 3° in patients with normal femoral torsion to 17° in patients with FAV upon physical examination [16]. Furthermore, the more significant the increase in femoral torsion, the greater the differences observed between the two methods [16]. In other words, the differences between the two methods increase progressively with the increase in femoral torsion, and the relationship between the two methods are trigonometric and not linear [16]. This must be considered especially when planning a rotational osteotomy in patients with severe femoral torsional abnormalities to avoid mistakes in preoperative planning. In summary, the fact that there is no consensus [10,11] as to how to measure torsion leaves the orthopedic surgeon in doubt about the confirmation of the diagnosis and, more importantly, in doubt about the surgical planning. 

Secondly, we should know where the torsion exists before we can decide where it should be corrected. In theory, the ideal would be to perform the osteotomy at the site where the deformity originates. However, torsion is normally measured as the angle between the proximal and the distal joint axes, so all that can be said is that it exists somewhere between the two reference axes. It seems that both the femoral neck and femoral shaft contribute to femoral tension [17,18,19,20]. Therefore, it is acceptable to perform the osteotomy at the proximal, mid-diaphyseal, or supracondylar level. Winkler et al. [21] have shown that increased external tibial torsion is an infratuberositary deformity and is not correlated with a lateralized position of the tibial tuberosity. Based on this finding, rotational tibial osteotomy should be performed infratuberositary. Since torsion is a measure of transverse plane alignment, any osteotomy that alters torsion must be made exactly perpendicular to the long axis of the bone to avoid creating a deformity in another plane.

The third question we ask ourselves is how much torsion should be corrected. The medical literature does not give us an answer to this question. If we do not correct the torsion enough, the pain will persist, and if we correct more than necessary the pain will persist. It has been shown that internal femoral rotational malalignment greater than or equal to 10° may provoke AKP [22,23]. It is logical to correct to the normal value in the population, but with no consensus of measurement, the value in the normal population is unknown. 

A fourth reason for uncertainty when we face this type of patient with torsional alterations is the knowledge that there are patients with obvious torsional anomalies who are completely asymptomatic. The only explanation for this finding is that their level of activity is low enough not to apply sufficient stress to bone and or peripatellar soft tissues. We must take note that an abnormal anatomy is only a risk factor for developing AKP [24]. However, we do not know the time and magnitude of stress on bone and/or soft tissues, which are necessary to provoke the physiopathological mechanisms that lead to pain and make a person become a patient. 

A fifth cause of uncertainty exists when abnormal torsion is found in both the femur and the tibia. Does abnormal femoral or abnormal tibial torsion contribute more to the genesis of AKP or does one add or multiply the effect of the other? From an anatomical standpoint, the best option is to treat the site of the abnormality. Therefore, the best option to treat a patient with combined excessive femoral internal torsion and excessive external tibial torsion would be a combination of a rotational femoral and a tibial osteotomy. Another option would be to operate on the bone with the greatest variance from normal. Currently, there is no scientific evidence to justify one or the other option.

Lastly, there is the concern about the myth that changing the tibial tuberosity (TT) can correct a torsional limb deformity. In fact, TT osteotomy (TTO) has undoubtedly overshadowed rotational osteotomy. At this point, it would be interesting to make some observations on the surgery of the TT in patients with torsional abnormality. Mani et al. [25] have demonstrated that TT medialization increases tibial external rotation. Therefore, greater AKP could triggered if we perform a medialization of the TT in patients with excessive external tibial torsion. Moreover, Tensho et al. [26] have shown that TT-TG distance is affected more by knee rotation than by TT malposition. For that reason, the measurement of the TT-TG distance in patients with torsional abnormalities is not reliable. Finally, Franciozi et al. [27] have seen diminished results from TTO in patients with increased FAV. Therefore, the best available evidence supports not performing TTO in patients with torsional abnormalities. On the other hand, the frequency and types of major complications seen in rotational osteotomy surgery are similar to those of the TTO (3.3% vs. 3%).
